# ROR1 CAR-T cells and ferroptosis inducers orchestrate tumor ferroptosis via PC-PUFA2

**DOI:** 10.1186/s40364-025-00730-0

**Published:** 2025-01-23

**Authors:** Dan Li, Wenjie Zhang, Ruiheng Wang, Shufeng Xie, Yixin Wang, Wanxin Guo, Zixuan Huang, Chaoqun Lu, Liang Shan, Han Liu, Lifang Ma, Xumin Hou, Zhenshu Xu, Jiayi Wang

**Affiliations:** 1 Department of Clinical Laboratory Medicine, Shanghai Chest Hospital, Shanghai Jiao Tong University School of Medicine, Shanghai, 200030 China; 2 Fujian Institute of Hematology, Fujian Provincial Key Laboratory of Hematology,, Fujian Medical University Union Hospital, Fuzhou, 350001 China; 3 Shanghai Institute of Hematology, State Key Laboratory of Medical Genomics, Rui Jin Hospital, School of Medicine, School of Life Sciences and Biotechnology, National Research Center for Translational Medicine at Shanghai, Shanghai Jiao Tong University, Shanghai, 200030 China; 4 Shanghai Institute of Thoracic Oncology, Shanghai Chest Hospital, Shanghai Jiao Tong University School of Medicine, Shanghai, 200030 China; 5 Hospital’s Office, Shanghai Chest Hospital, Shanghai Jiao Tong University School of Medicine, Shanghai, 200030 China

**Keywords:** Lung cancer, ROR1, CAR-T, Ferroptosis, PC-PUFA2

## Abstract

**Background:**

Lung cancer, particularly non-small cell lung cancer (NSCLC), has high recurrence rates and remains a leading cause of cancer-related death, despite recent advances in its treatment. Emerging therapies, such as chimeric antigen receptor (CAR)-T cell therapy, have shown promise but face significant challenges in targeting solid tumors. This study investigated the potential of combining receptor tyrosine kinase-like orphan receptor 1 (ROR1)-targeting CAR-T cells with ferroptosis inducers to promote ferroptosis of tumor cells and enhance anti-tumor efficacy.

**Methods:**

RNA-seq data and immunofluorescence analysis of relapsed NSCLC patient samples were used to explore ROR1 expression. In addition, ROR1-targeting CAR-T cells were developed to assess cytotoxic activity against ROR1^+^ tumor cells, and the effect of cytokine stimulation on their efficacy was evaluated. Lipidomics, immunofluorescent histochemistry, and western blotting were used to explore the observed effects. Ferroptosis indicators, including levels of reactive oxygen species, were used to detect the combined effect of CAR-T cells and ferroptosis-inducing drugs. Finally, tumor-bearing mice were used to validate the in vivo efficacy of the combination therapy strategy.

**Results:**

Tumor cells treated with ferroptosis inducers showed increased sensitivity to Interferon gamma (IFN-γ) secreted by ROR1 CAR-T cells. Furthermore, ROR1 CAR-T cells enhanced the production of phosphatidylcholine with diacyl-polyunsaturated fatty acid tails (PC-PUFA2) by working in tandem with IFN-γ. This enhancement promoted the expression of acyl-CoA synthetase long chain family member 4 (ACSL4), which in turn strengthened the overall anti-tumor response.

**Conclusions:**

Combining ROR1 CAR-T cells with ferroptosis inducers enhanced anti-tumor efficacy in NSCLC by promoting ferroptosis through increased lipid peroxidation.

**Supplementary Information:**

The online version contains supplementary material available at 10.1186/s40364-025-00730-0.

## Introduction

Lung cancer has been the leading cause of cancer-related death since the 1950s [1]. Despite significant advances in the treatment of non-small cell lung cancer (NSCLC) over the past two decades, particularly with the introduction of small molecule tyrosine kinase inhibitors and immunotherapies offering unprecedented survival benefits [2], resistance and relapse following standard treatments remain major challenges [3, 4]. The recurrence rate for patients with stage I or II NSCLC is approximately 20%, while stage IIIA patients face a 41% likelihood of recurrence or resistance within the first year [5, 6]. Currently, no effective therapies exist for late-stage lung cancer, underscoring the urgent need for novel therapeutic strategies.

The advent of chimeric antigen receptor (CAR)-T cell therapy has brought renewed hope to patients with refractory tumors. Several CAR-T cell products targeting CD19 and BCMA antigens have been approved for clinical use [7–9]. However, CAR-T cell therapy faces significant obstacles in the treatment of solid tumors [10], which comprise over 90% of all cancers [11]. Solid tumors are often characterized by dense stromal components, including tumor-associated fibroblasts and an extensive vasculature, creating a physical barrier that impedes immune cell infiltration [12–14]. To overcome these challenges, combination therapies have been developed to convert “cold” tumors into “hot” tumors, which are more susceptible to immune responses. This conversion is a promising strategy for enhancing anti-tumor efficacy in patients with relapsed and refractory disease [15].

A key reason for treatment failure in cancer is defective cell death mechanisms in tumor cells [16]. Tumor cells exhibit a higher demand for iron than non-tumor cells and are therefore more vulnerable to iron-catalyzed necrosis, a form of non-apoptotic cell death known as ferroptosis [17–19]. A novel approach to cancer treatment by inducing ferroptosis to exploit this vulnerability can eliminate tumor cells and curb the proliferation of resistant cells [20, 21]. Whether ferroptosis inducers can reverse resistance in lung cancer cells and enhance the sensitivity of CAR-T therapy remains to be determined.

Receptor tyrosine kinase-like orphan receptor 1 (ROR1), a member of the type I receptor tyrosine kinase (RTK) family, has emerged as a highly promising therapeutic target [22]. Notably, ROR1 is highly expressed in various tumor cell types but exhibits minimal expression in healthy adult tissues, providing a strong basis for therapeutic exploitation [23, 24]. 42% of lung adenocarcinoma patients express ROR1, with 38% exhibiting high levels of expression [22], making ROR1 a viable target for the treatment of NSCLC.

This study addressed the challenge of treating NSCLC by combining ROR1-targeting CAR-T cells with ferroptosis inducers. Using this approach, we aimed to reverse tumor cell resistance while increasing the sensitivity of tumor cells to Interferon gamma (IFN-γ). Furthermore, we hypothesized that CAR-T cell stimulation will sensitize tumor cells to ferroptosis via acyl-CoA synthetase long chain family member 4 (ACSL4) and phosphatidylcholine with diacyl-polyunsaturated fatty acid tails (PC-PUFA2) pathways, providing dual mechanisms of anti-tumor activity. Our findings validate the efficacy of targeting ROR1 in solid tumors and offer new therapeutic hope for patients with recurrent and refractory NSCLC.

## Materials and methods

### Dataset analysis

ROR1 expression data and clinical survival data of NSCLC patients were acquired from the Gene Expression Omnibus (GEO) database (GSE135222). Tissue samples obtained from patients prior to treatment were utilized to assess ROR1 expression levels, and subsequently, the sensitivity of these patients to immune checkpoint blockade (ICB) therapy was analyzed.

### Patient samples

Tissue samples were collected from patients diagnosed with NSCLC at Shanghai Chest Hospital, following the receipt of written informed consent. A portion of each tumor biopsy was formalin-fixed (using 10% formalin for 48 h) and subsequently embedded in paraffin for subsequent immunofluorescence studies. The remainder of each tumor sample was manually minced using a sterile scalpel and then enzymatically digested with 1 mg/mL Type I collagenase for 4 h in a gentleMACS Dissociator, (Miltenyi Biotec). Dissociated cells were cultured in 10-cm dishes containing Dulbecco’s Modified Eagle’s Medium (DMEM) supplemented with 10% fetal bovine serum (FBS).

### Reagents

The following reagents were used: RSL3 [(1 S,3R)-RSL3, MedChemExpress, HY-100218 A], ML210 (MedChemExpress, HY-10003), erastin (MedChemExpress, HY-15763), Fer-1 (ferrostatin-1, Selleck, S7243), MG132 (MedChemExpress, HY-13259), Baf-A1 (Bafilomycin A1, MedChemExpress, HY-100558), decitabine (Selleck, S1200), paclitaxel (Selleck, S1150), rapamycin (Selleck, S1039), cisplatin (Selleck, S1166), and etoposide (Selleck, S1225).

### DNA constructs and lentivirus production

The ROR1 CAR construct included a signal peptide, and anti-ROR1 single-chain fragment variable, hinge, transmembrane domain, 4-1BB, and CD3ζ domains. The construct was cloned into a pCDH-T2A-mRuby2 lentiviral vector, and lentivirus produced by co-transfecting 293T cells with psPAX2 and pMD2.G plasmids using ProFection^®^ Mammalian Transfection System Calcium Phosphate (Promega, E1200). Lentivirus-containing supernatants were harvested at 24 and 48 h post-transfection, filtered, and used for T-cell transduction.

CRISPR guide RNAs targeting *IFNGR1* and *ACSL4* were designed using the online tool provided by the Zhang laboratory at http://crispr.mit.edu/. Following synthesis, guide RNAs were cloned into the LentiCRISPR v2 vector (Addgene, 52961). The sequences of the sgRNAs are detailed in Supplementary Table S1.

### Generation of hCAR-T cells and culture conditions

Peripheral blood mononuclear cells were isolated from healthy donors by density gradient centrifugation. T cells were purified using the EasySep Human T Cell Isolation Kit (STEMCELL Technologies, 17951) and activated with CD3/CD28 Dynabeads (Thermo Fisher Scientific, 11141D) following ethical and safety guidelines.

Primary T cells were cultured in RPMI 1640 medium (Gibco, 11875093) supplemented with 10% FBS, 100 IU/ml human IL2 (PeproTech, 200-02) and 0.05 mM β-mercaptoethanol (Gibco, 21985023).

### Flow cytometry

ROR1 and IFNGR1 expression on NSCLC cell lines and primary cells was analyzed by flow cytometry. Single-cell suspensions were stained with an anti-ROR1 monoclonal antibody (Biolegend, 357806) or a phycoerythrin-conjugated anti-human CD119 (IFN-γ R α chain) antibody (Biolegend, 308703) for 30 min on ice. Data from 10,000 cells were collected and analyzed using FlowJo software.

Untransduced T cell and CAR-T cell surface receptor expression was analyzed by flow cytometry (BD Fortessa) after labeling samples with specific antibodies. The antibodies used were as follows: PE/Cyanine5 anti-human CD3 (Biolegend, 317356), Brilliant Violet 421 anti-human CD4 (Biolegend, 344632), Brilliant Violet 510 anti-human CD8 (Biolegend, 344732), APC anti-human CD69 (Biolegend, 310910), Spark PLUS UV39 anti-human CD45RA (Biolegend, 304190), and APC anti-human CD62L (Biolegend, 385106).

### Reactive oxygen species (ROS) and peroxidation measurement

Lipid peroxidation was quantified using BODIPY 581/591 C11 (Invitrogen, D3861) at a 1:2000 dilution in serum-free medium. Cells were incubated with the probe at 37 °C in the dark for 20–30 min, washed with PBS, and analyzed by flow cytometry. Data were processed using FlowJo software.

### Real-time cell analysis

The cytolytic activity of ROR1 CAR-T cells was evaluated using the xCELLigence Real-Time Cell Analysis (RTCA) MP system (Agilent, San Diego, CA) according to the manufacturer’s protocol. Target cells were seeded at a density of 10,000 cells/well and incubated at 37 °C. After 4 h, untransduced T cells or ROR1 CAR-T cells were added at a 1:1 E: T ratio with or without RSL3. The cell index was collected at 30-minute intervals for 36 h and reflected the quality of live cell adhesion, total cell number and cell morphology.

### Cell viability and cytokine secretion

Cell viability assays were performed by incubating untransduced T cells, CAR-T cells, PC-PUFA2 [PC (22:6_22:6)-CH3, Cayman, 10733], palmitoleic acid (MedChemExpress, HY-W011873) or IFN-γ (GenScript, Z02986) with NCI-H1299 or primary cells. Target cells were seeded at a density of 10,000 cells/well in 96-well plates. Target cell viability in effector-target co-culture experiments (1:1 E: T ratio) was quantified using the ONE-Glo Luciferase Assay Kit (Promega, E6120). Firefly luciferase reporter gene expression was assessed in viable luciferase-tagged NCI-H1299 and primary tumor cells. The CellTiter-Glo Luminescent Cell Viability Assay kit (Promega, G7571) was employed in other studies to assess tumor cell viability. Tumor cell death based on cell viability was calculated by subtracting the percentage of viable cells.

To analyze the secretion of IFN-γ and TNF-α pro-inflammatory cytokines by CAR-T cells into the supernatant, target cells were seeded in 24-well plates at 1 × 10^5^ cells per well. Supernatant was collected 24 h after co-culture with or without CAR-T cells and ferroptosis inducer at an effector: target ratio of 1:1. IFN-γ and Tumor necrosis factor alpha (TNF-α) were detected using ELISA kits (Abclonal, RK000015, RK00030) according to the manufacturer’s protocol.

### Relative quantitative lipidomics

NCI-H1299 cells were collected after treatment with RSL3, ROR1 CAR-T cells, or their combination. Lipids were extracted using a methyl-tert-butyl ether method. Reverse phase chromatography was selected for separation using a CSH C18 column (1.7 μm, 2.1 mm× 100 mm, Waters). The lipid extracts were re-dissolved in 200 µL 90% isopropanol/acetonitrile, centrifuged at 14,000 × g for 15 min, and 3 µL of sample was injected. Solvent A was acetonitrile–water (6:4, v/v) containing 0.1% formic acid and 0.1 mM ammonium formate and solvent B was acetonitrile–isopropanol (1:9, v/v) containing 0.1% formic acid and 0.1 mM ammonium formate. The initial mobile phase was 30% solvent B at a flow rate of 300 µL/min. This was held for 2 min, and then linearly increased to 100% solvent B in 23 min, followed by equilibration with 5% solvent B for 10 min. Mass spectra were acquired using a Q-Exactive Plus mass spectrometer in positive and negative mode.

### Immunofluorescence

An anti-ROR1 antibody (Abcam, ab111174) was used for immunofluorescence imaging of ROR1 in primary patient-derived samples. For immunofluorescence imaging of Transferrin Receptor 1 (TfR1), cells were seeded on round coverslips in 24-well plates at 1 × 10^5^ cells per well and cultured overnight. Cells were treated with 100 µΜ PC-PUFA2 for 6 h. Cells were then fixed with 4% paraformaldehyde for 20 min, blocked using 10% normal goat serum for 1 h, and stained with an anti-TfR1 antibody (CST, 13113T) at 1:100 dilution overnight. Cells were then stained with secondary antibodies at 1:200 dilution for 1 h at room temperature. At least two biological replicates were performed for all immunofluorescence assays.

### Image collection and processing

All slides were imaged using a Zeiss LSM800 confocal microscope with a 63×/1.40 Oil DIC objective. All imaging parameters, including laser power, scanning speed, and scanning area, remained constant for each experiment. Images were captured randomly across each well to ensure unbiased data collection. Fluorescence intensity was quantified using Image J software.

### Western blot

Approximately 1 × 10^6^ cells were collected and washed with PBS. The cell pellet was lysed in 100 µL Cell Complete Lysis Buffer for Western and IP (Beyotime, P0037) on ice for 10 min. The lysate was then centrifuged at 12,000 × g for 10 min at 4 °C. The supernatant was collected, quantified, diluted with 3× blue loading buffer containing 125 mM DTT, and incubated at 99 °C for 10 min. Equal amounts of protein were loaded into each lane of a NuPAGE 10% Bis-Tris gel, separated, and subsequently transferred onto a PVDF membrane (Millipore) using an electrophoretic semi-dry western blot transfer system. Membranes were blocked with PBS blocking buffer for 1 h at room temperature and incubated with a primary antibody overnight at 4 °C. After three washes in PBS-T, the membranes were incubated with secondary antibodies diluted 1:2,000 for 1 h at room temperature. Finally, the membranes were washed three more times in PBS-T and imaged using the GE Amersham Imager 600 system. The primary antibodies used were anti-ROR1 (CST, 16540), rabbit anti-ACSL4 monoclonal (Abclonal Technology, A20414), and anti-beta Actin monoclonal (Proteintech, 2D4H5).

### NSCLC metastasis model

Female NOD/ShiLtJGpt-*Prkdc*^*em26Cd52*^*Il2rg*^*em26Cd22*^/Gpt (NCG) mice, aged 6–8 weeks, were purchased from GemPharmatech. The mice were engrafted with the NCI-H1299-GL cell line expressing luciferase via intravenous injection (3 × 10^6^ cells per mouse). Tumor engraftment was confirmed using bioluminescence imaging prior to any treatment, ensuring similar tumor burdens across all mice. The engrafted mice were randomly divided into vehicle or ferroptosis inducer groups, and then treated with intravenous injections of 3 × 10^6^ engineered CAR-T cells.

### Statistical analysis

Kaplan-Meier survival curves were generated and compared between the responsive and non-responsive patients using the log-rank test. All statistical analyses were performed using R version 4.0.0 (R-project.org) and its associated packages. Statistical significance was set at *p* < 0.05.

For group comparisons with normal distributions and continuous endpoints, data are presented as the mean ± standard error of the mean (SEM). Student’s t-test was used to compare two independent groups. A linear mixed model was applied to account for the variance-covariance structure caused by repeated measures from the same subjects. Survival functions were estimated using the Kaplan-Meier method, and comparisons between groups were made using the log-rank test. A *p* value < 0.05 was defined as statistically significant. GraphPad 9 was used for statistical analyses.

## Results

### ROR1 is highly expressed in ICB-resistant NSCLC tumors and correlates with poor patient survival

**Fig. 1:**
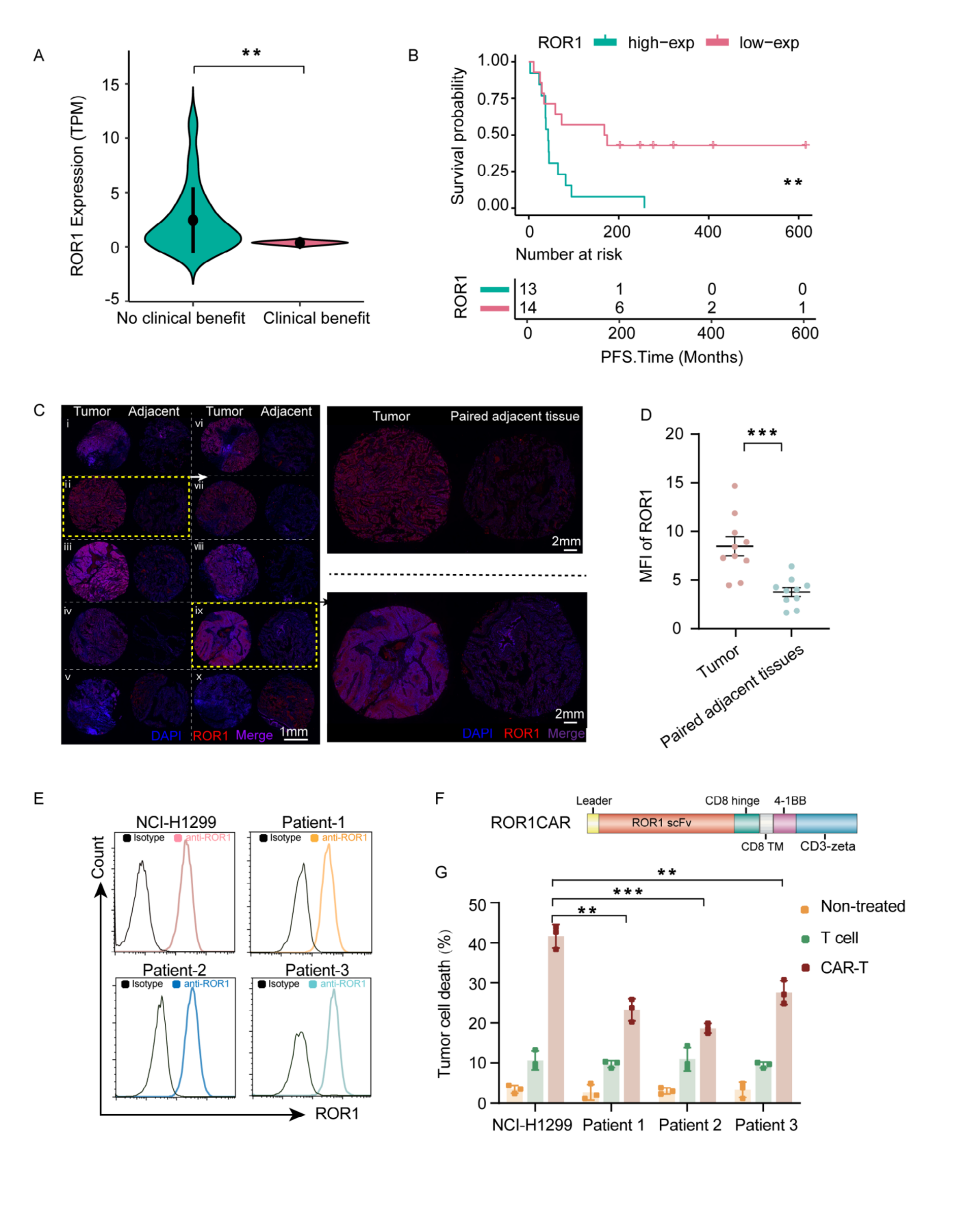
Evaluation of ROR1 as a therapeutic target in recurrent lung cancer patients. (**A**) *ROR1* expression in PD-1/PD-L1 responding and non-responding cohorts. (**B**) Correlation of survival outcomes with *ROR1* expression following PD1 treatment. Statistical significance was determined using median-rank analysis. (**C**–**D**) Detection of ROR1 expression in tumor tissues from recurrent NSCLC patients. (**E**) ROR1 expression levels on the surface of NCI-H1299 cell lines and primary cells derived from three recurrent patients. (**F**) Schematic representation of the ROR1 CAR molecular sequence. (**G**) Percentage of target cell death following a 24 h co-culture with ROR1 CAR-T cells or untransduced T cells (*n* = 3). Statistical significance: n.s. (*p* > 0.05); *p < 0.05; **p < 0.01; ****p < 0.0001

ROR1 expression in tumor cells is linked to poor prognosis in several cancers [22]. Analysis of RNA-seq data from GSE135222 (from 27 NSCLC patients treated with anti-PD1/PD-L1) revealed significantly higher *ROR1* mRNA levels in patients resistant to ICB than in patients who benefit from ICB treatment (*p* < 0.01) (Fig. 1A). Additionally, elevated *ROR1* expression was associated with shorter overall survival in these cases (Fig. 1B). These clinical findings indicate that ROR1 is a promising therapeutic target for patients with ICB-resistant NSCLC. To determine whether the observed increase in *ROR1* mRNA levels corresponded to higher protein levels, immunofluorescence staining was conducted on NSCLC tumor samples and adjacent normal tissues. Of the 10 tumor samples obtained from ICB-resistant patients, eight (80%) were positive for ROR1, with five samples showing strong positivity and three showing weak positivity, while nonmalignant cells in adjacent normal tissues exhibited minimal ROR1 expression (Fig. 1C–D, Figure S1A). The detailed clinical features of these patients are summarized in Table S2.

### ROR1 CAR-T cells specifically target ROR1-positive cancer cells

To identify suitable targets for functional validation of the engineered CAR-T cells, ROR1 expression was confirmed in both the NCI-H1299 cell line and primary NSCLC cells using flow cytometry (Fig. 1E). These findings prompted the development of an immunotherapy targeting ROR1 for NSCLC. The anti-ROR1 single-chain variable fragment (ROR1 scFv) was incorporated into a second-generation CAR construct, which included a CD8 signal sequence, a CD8 hinge, a CD8 transmembrane domain, a 4-1BB costimulatory domain, and a CD3ζ intracellular signaling domain (Fig. 1F, Figure S1B). This construct was then transduced into primary T cells using lentiviral vectors. For functional validation, ROR1-positive tumor cells were selected. Lung cancer cells were co-cultured with either unmodified T cells or ROR1 CAR-T cells for 24 h at the ratio of 1:1. T cell activation was then assessed by ELISA determination of IFN-γ secretion (Figure S1C). ROR1 CAR-T cells exhibited significantly higher cytotoxicity against the NCI-H1299 cell line, while demonstrating limited activity against primary tumor cells from patients who had undergone multiple rounds of treatment (Fig. 1G).

### Validation of drugs that influence CAR-T cell cytotoxicity

**Fig. 2:**
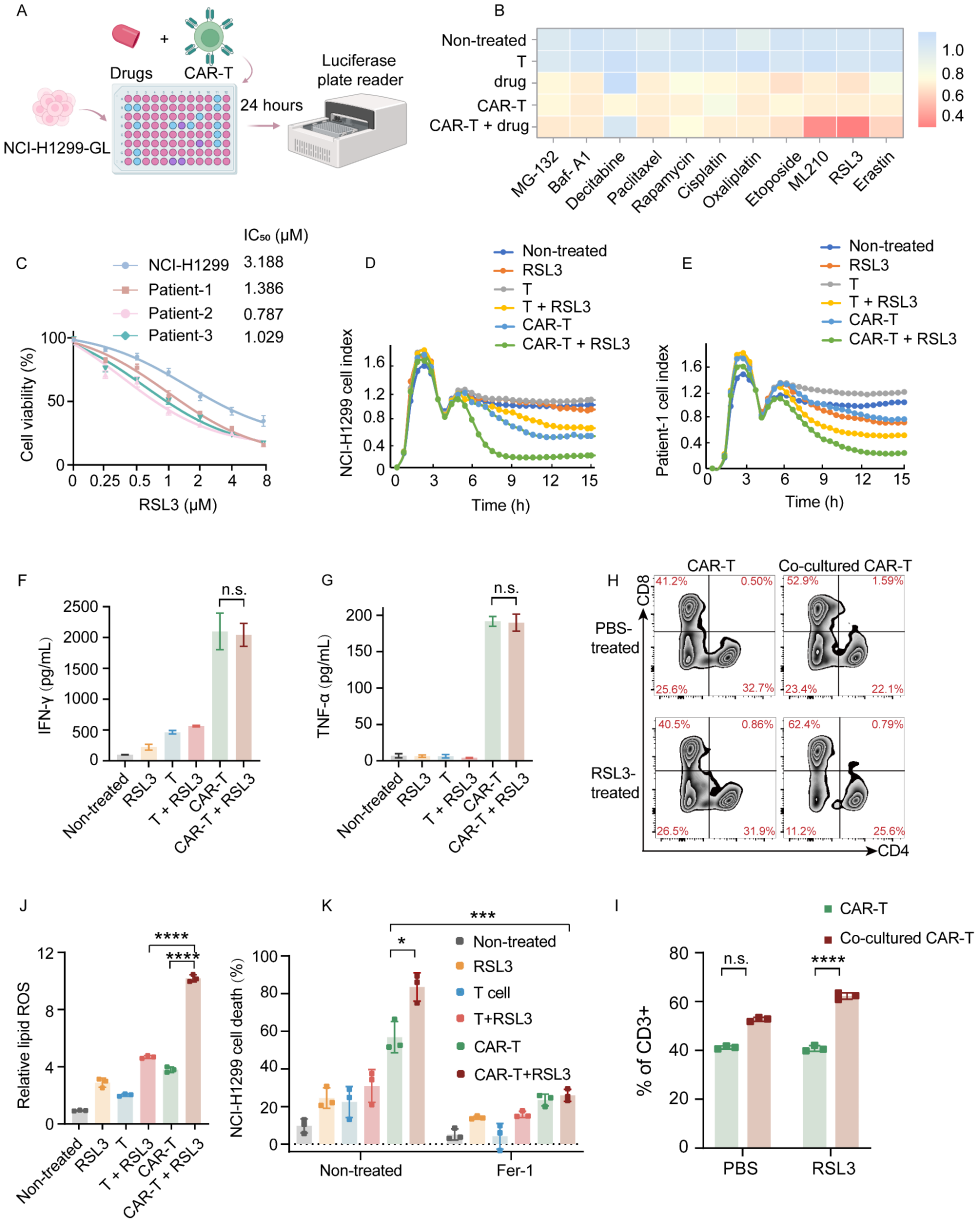
Validation of key drugs impacting CAR-T cell cytotoxicity. (**A**) Schematic illustration of the screen for candidate drugs that enhance CAR-T cell cytotoxicity. Figure generated using BioRender (https://biorender.com/). (**B**) Heatmap showing the activity of NCI-H1299-GL cells treated with various drugs and in combination with CAR-T cells using the screening system (*n* = 3). (**C**) Viability of lung cancer cells treated with varying concentrations of RSL3 (*n* = 3). (**D**–**E**) Tumor cell killing efficacy of combination therapy measured by RTCA (*n* = 3). (**F**–**G**) ELISA measurement of IFN-γ and TNF-α levels in the supernatants from the co-culture of ROR1 CAR-T and NCI-H1299 cells (*n* = 3). (**H**–**I**) CAR-T cells were co-cultured with H1299 cells in the presence or absence of RSL3 for 48 h. The CAR-T cells were then harvested, and the relative proportions of CD4 and CD8 cells within the CD3-positive cell population were determined using flow cytometry. (**J**) Lipid peroxidation in NCI-H1299 cells treated with or without ROR1 CAR-T cells and RSL3 was detected using C11-BODIPY 581/591 staining. Relative mean fluorescence intensity (MFI) of oxidized C11-BODIPY 581/591 is presented as the mean ± SEM (*n* = 3). (**K**) Percentage NCI-H1299-GL cell death in the co-culture system with or without the ferroptosis inhibitor, Fer-1 (*n* = 3). Statistical significance: n.s. (*p* > 0.05); *p < 0.05; **p < 0.01; ****p < 0.0001

To identify small-molecule drugs that influence CAR-T-cell cytotoxicity, we performed a cell death inducer screen using a co-culture assay with ROR1-directed CAR-T cells and NCI-H1299 cells expressing green fluorescent protein (GFP) and luciferase (NCI-H1299-GL cells) (Fig. 2A). The drugs screened included several classical cell death inducers, such as apoptotic modulators, cell cycle inhibitors, DNA damage and autophagy inducers, as well as several ferroptosis inducers. We then exposed NCI-H1299-GL cells to those compounds that showed promise at six different concentrations for 24 h, both alone and in the presence of CAR-T cells, and measured target cell viability using the luciferase assay. The drugs that most significantly increased CAR-T cell cytotoxicity were RSL3 and ML210 (Fig. 2B), both of which are glutathione peroxidase 4 (GPX4) inhibitors. The IC_50_ of RSL3 in NCI-H1299 cells was then compared with different donor cells, which demonstrated that primary tumor cells were the most sensitive (Fig. 2C). To confirm the screening findings, we also tested the two drugs on CAR-T cells and primary lung cancer cells using the real-time cell analysis system. Notably, even at an RSL3 concentration as low as 0.5 µM, we obtained consistent results with cells from different donors, indicating that the assay is robust and that the drug effects observed are consistent with those from the initial screen. (Fig. 2D–E and Figure S2A–B).

We then performed ELISA evaluation of T cell activation markers, IFN-γ and TNF-α in the supernatant. The addition of the drugs did not result in a significant change in T-cell activation (Fig. 2F–G). To assess the impact of the drugs on T cell phenotypes, we detected the CAR-T cell positive rate (Figure S2C-D) and the CD8^+^/CD4^+^ CAR-T cell ratio after co-culture with tumor cells with or without RSL3. After co-culture with NCI-H1299 cells for 48 h, there was a significant increase in the proportion of CD8^+^/CD4^+^ CAR-T cells in both the untreated and treated RSL3 groups (Fig. 2H–I). Increased CD8^+^ CAR-T cell numbers indicate a more robust cytotoxic response. Furthermore, there was no notable distinction in the CAR-T cell activation marker, CD69, between the RSL3-treated and untreated groups following the co-culture with tumor cells (Figure S2E).

For T cell adoptive cell therapy to achieve robust efficacy, it is preferable to have a diverse population of memory T cell phenotypes that can integrate cytotoxic capabilities with long-term persistence and immunological memory [25, 26]. Memory T cells are classified on the basis of specific surface markers: effector memory T cells (Tem) as CD62L^−^CD45RA^−^, central memory T cells (Tcm) as CD62L^+^CD45RA^−^, and naive or stem cell-like memory T cells (Tn/Tscm) as CD62L^+^CD45RA^+^. Upon co-culturing CAR-T cells with NCI-H1299 cells, the proportion Tcm cells increased, rising from 40 to 67.5%. This increase was considerably more pronounced than that observed in the T cell group, which only saw a modest rise from 42.9 to 50.4%. Tcm shifts were broadly consistent even in the presence of RSL3 (Figure S2F). Tcm cells are the primary reservoir of proliferating T cells and are responsible for immunological memory. Therefore, there is no significant effect on T cell phenotype and function in the presence of low concentrations of RSL3.

However, increased ROS levels were observed in tumor cells following treatment (Fig. 2J). The death of tumor cells was also inhibited by Fer-1, indicating a potential mechanism for the enhanced cytotoxicity (Fig. 2K).

### ROR1 CAR-T and RSL3 coordinately induce tumor cell ferroptosis via PC-PUFA2

**Fig. 3:**
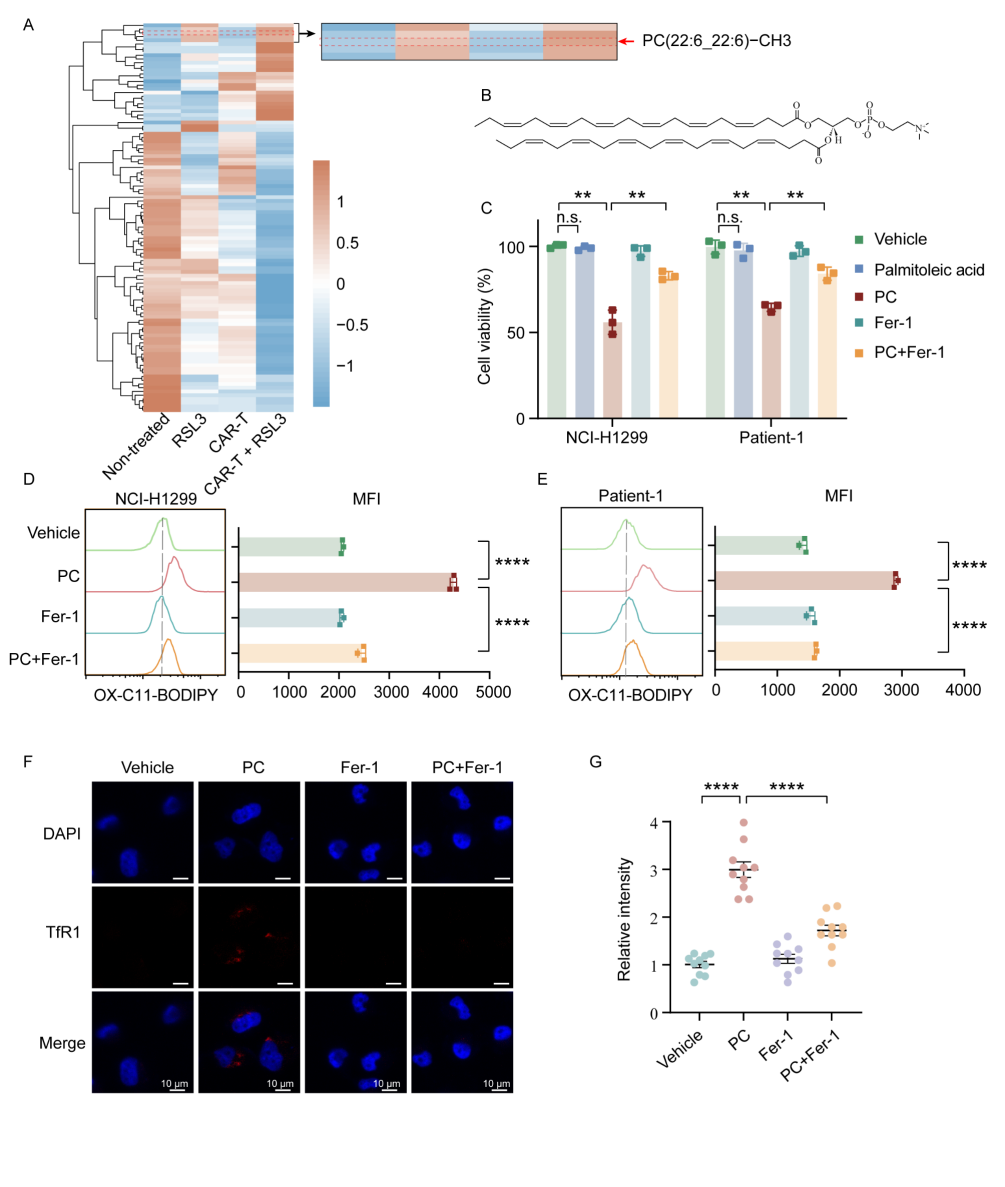
ROR1 CAR-T and RSL3 cooperatively induce tumor cell ferroptosis via PC-PUFA2. (**A**) Lipidomic profiles of NCI-H1299 cells treated with control, CAR-T, RSL3, and combination treatments (*n* = 5). (**B**) Structure of PC-PUFA2. (**C**) NCI-H1299 and patient-1 cells were co-treated with 50 µM PC-PUFA2 with or without 10 µM Fer-1 for 24 h. 50 µM palmitoleic acid was added as a control lipid. CellTiter-Glo reagent was added to the culture, and the viability of tumor cells was subsequently assessed by measuring luminescence (*n* = 3). Data are presented as the mean ± SEM. (**D**–**E**) Lipid peroxidation detected by C11-BODIPY 581/591 staining in NCI-H1299 and patient-1 cells treated with 50 µM PC-PUFA2 with or without 10 µM Fer-1 for 4 h. Relative mean fluorescence intensity (MFI) of oxidized C11-BODIPY 581/591 plotted as the mean ± SEM (*n* = 3). (**F**–**G**) Immunofluorescence of TfR1 expression in NCI-H1299 cells treated with vehicle, 10 µM Fer-1, or 50 µM PC (22:6_22:6) with or without 10 µM Fer-1 for 4 h. Scale bars, 10 μm. Relative intensity of TfR1 membrane staining compared with vehicle is plotted as the mean ± SEM (*n* = 10). Statistical significance: n.s. (*p* > 0.05); *p < 0.05; **p < 0.01; ****p < 0.0001

The ferroptosis inducers, RSL3 and ML210, function by inhibiting the activity of GPX4, an enzyme responsible for reducing phospholipid hydroperoxide (PL-OOH) to its alcohol form (PL-OH) [27, 28]. To explore the mechanism by which RSL3 enhances tumor cell death following ROR1 CAR-T cell treatment, we harvested NCI-H1299 cells and performed lipidomic analyses. As shown in Fig. 3A, levels of PC (22:6_22:6)-CH3 (PC-PUFA2) increased following the combined treatment with CAR-T cells and RSL3. Specifically, PC-PUFA2 demonstrated a markedly higher propensity to induce ferroptosis [29]. A specific PC-PUFA2 was elevated in the combination treatment group compared with the RSL3 treatment group, but not in the CAR-T-only group (Fig. 3A–B, Table S3).

To further investigate the role of PC in ferroptosis regulation, we treated the NCI-H1299 cell line and primary patient-1 tumor cells with PC-PUFA2 and assessed its effect on cell viability. A notable reduction in cell viability was observed following PC-PUFA2 treatment (Fig. 3C). The role of PC-PUFA2 in ferroptosis induction was confirmed by the rescue of cell viability in the presence of the ferroptosis inhibitor, Fer-1. Additionally, PC-PUFA2 treatment significantly increased lipid peroxide accumulation in both NCI-H1299 and patient-1 tumor cells, as detected by the fluorescent probe C11-BODIPY, which exhibits a peak shift upon oxidation by lipid peroxides. This lipid peroxidation was effectively inhibited when cells were co-treated with Fer-1 (Fig. 3D–E). Moreover, NCI-H1299 cells treated with PC-PUFA2 exhibited a significant increase in the translocation of the ferroptosis marker, TfR1, to the plasma membrane. This effect was reversed upon co-treatment with Fer-1 (Fig. 3F–G). Collectively, these findings indicate that PC-PUFA2s are potent inducers of ferroptosis when combined with ROR1 CAR-T cells and RSL3.

### CAR-T cells enhance ferroptosis via the IFN-γ–ACSL4–PC-PUFA2 axis

**Fig. 4:**
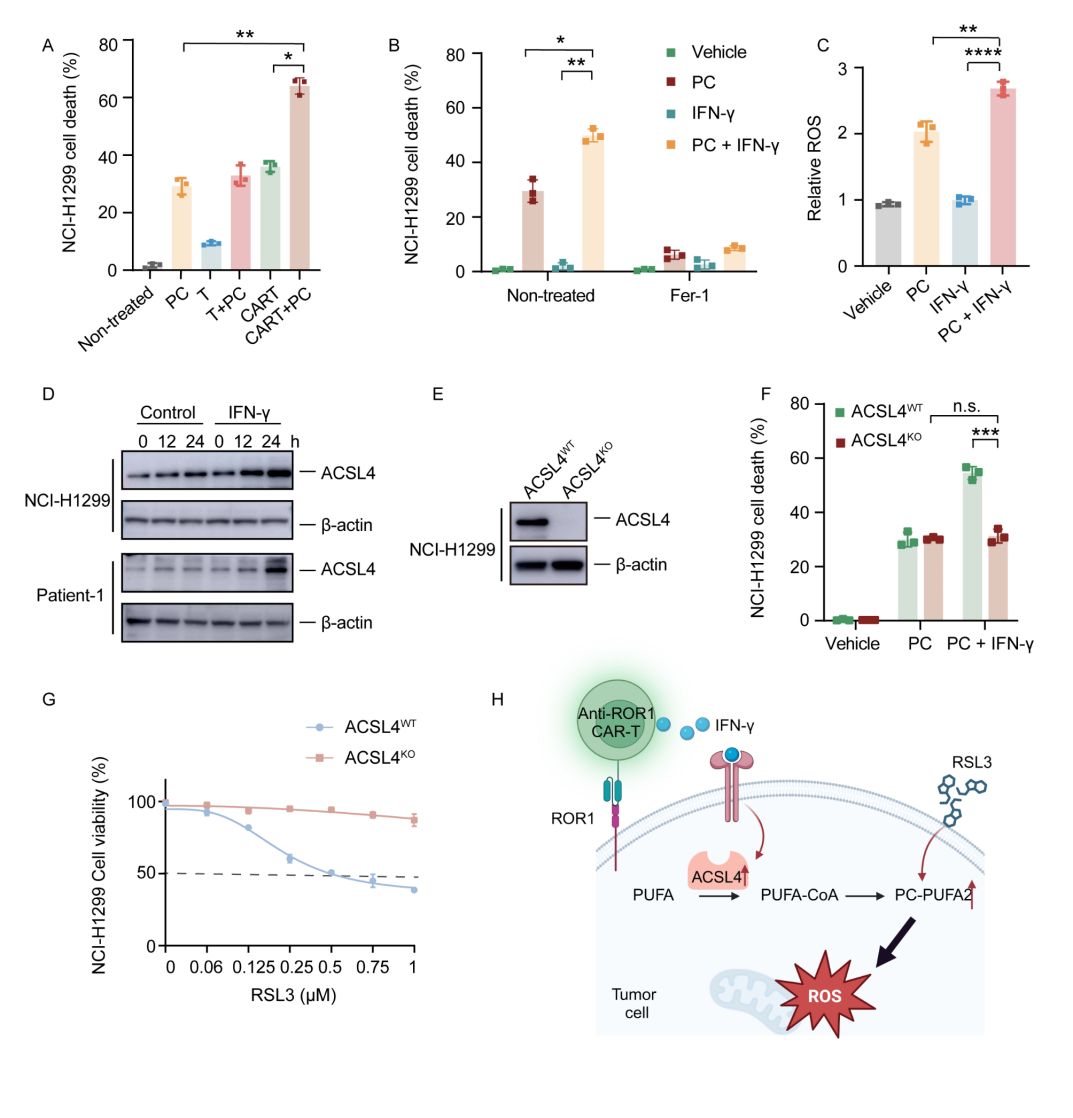
CAR-T cells promote ferroptosis via the IFN-γ–ACSL4–PC-PUFA2 pathway. (**A**) Percentage target cell death after co-culture for 24 h with ROR1 CAR-T cells or untransduced T cells with or without 50 µM PC-PUFA2 (*n* = 3). (**B**) NCI-H1299 cells treated with vehicle, 10 ng/ml IFN-γ, or 50 µM PC-PUFA2 with or without 10 µM Fer-1. Cell death was measured after 24 h (*n* = 3). (**C**) Relative mean fluorescence intensity of oxidized C11-BODIPY 581/591 compared with vehicle, presented as the mean ± SEM (*n* = 3). (**D**) Western blot analysis of ACSL4 levels in NCI-H1299 cells treated with 10 ng/ml IFN-γ for the indicated times. (**E**) ACSL4 knockout in NCI-H1299 cells confirmed by western blotting. (**F**) Cell death of NCI-H1299 ACSL4^wt^ and NCI-H1299 ACSL4^KO^ cells treated with 50 µM PC-PUFA2 with or without 10 ng/ml IFN-γ for 24 h (*n* = 3). (**G**) Cell viability of NCI-H1299 ACSL4^wt^ and NCI-H1299 ACSL4^KO^ cells treated for 10 h with RSL3 at the indicated concentrations (*n* = 3). (H) Schematic of the IFN-γ–ACSL4–PC-PUFA2 axis, generated using BioRender (https://biorender.com/). Statistical significance: n.s. (*p* > 0.05); *p < 0.05; **p < 0.01; ****p < 0.0001

We hypothesized that polyunsaturated fatty acids (PUFAs) can act together with CAR-T cells to promote tumor cell death. To validate this hypothesis, we measured the tumor cell-killing potential of CAR-T cells in both the NCI-H1299 cell line and primary tumor cells, with and without PC-PUFA2. As anticipated, PC-PUFA2 synergized with ROR1 CAR-T cells to induce significant cell death of both NCI-H1299 cells and primary tumor cells (Fig. 4A and Figure S3A). IFN-γ is released by CD8 + T cells and promotes lipid peroxidation and ferroptosis in tumor cells [30, 31]. Therefore, we focused on the interaction between IFN-γ and PC-PUFA2. To determine whether the combination of IFN-γ and PC-PUFA2 can increase tumor cell death by promoting ferroptosis, we treated the tumor cells with IFN-γ and PC-PUFA2 combined with Fer-1. The combination of IFN-γ and PC-PUFA2 induced significant cell death in both NCI-H1299 cells and primary tumor cells, which was rescued by Fer-1 (Fig. 4B, Figure S3B). Additionally, the IFN-γ and PC-PUFA2 combination increased lipid ROS levels in both NCI-H1299 cells and primary tumor cells (Fig. 4C, Figure S3C). These results indicate that CAR-T cells contribute to this effect by promoting tumor ferroptosis through IFN-γ secretion.

Next, we explored the mechanism by which IFN-γ and PC-PUFA2 induce tumor cell ferroptosis. Acyl-CoA synthetase long-chain family member 4 (ACSL4) activates arachidonic acid to arachidonyl-CoA, which is then esterified into phospholipids. Exogenous arachidonic acid enhances RSL3-mediated ferroptosis [32], while CAR-T cells engineered to secrete IFN-κ induce tumor ferroptosis via an IFNAR/STAT1/ACSL4 axis [33]. Consistent with these findings, IFN-γ increased ACSL4 levels in both NCI-H1299 cells and primary tumor cells (Fig. 4D). To investigate whether ACSL4 is a key enzyme in the ferroptosis induced by IFN-γ and PC-PUFA2, we knocked out (KO) ACSL4 in NCI-H1299 cells (Fig. 4E). ACSL4^KO^ cells showed resistance to combination therapy. However, there was no significant difference when treated with PC-PUFA2 (Fig. 4F). Similarly, ACSL4^KO^ NCI-H1299 cells exhibited resistance to RSL3 and ML210-mediated ferroptosis (Fig. 4G, Figure S3D). ACSL4 was shown to be an IFN-γ downstream enzyme.

IFN-γ engages with its receptors (IFNGRs) to activate downstream signaling [34]. To confirm the pivotal regulatory function of IFN-γ in the ACSL4–PC-PUFA2 axis, we genetically ablated Interferon gamma receptor 1 (IFNGR1) in NCI-H1299 cells, as depicted in Figure S3E. The IFNGR1 knockout cells showed a marked resistance to the combined treatment compared with wildtype NCI-H1299 cells (Figure S3F). Given that IFN-γ is typically produced by activated T cells, the simultaneous presence of PC-PUFA2 and IFN-γ can be achieved through the combination of CAR-T cells and RSL3, offering a potential mechanism to induce tumor cell ferroptosis within the tumor microenvironment (Fig. 4H).

### ROR1 CAR-T cells suppress tumor growth in a metastatic mouse model of human NSCLC

**Fig. 5:**
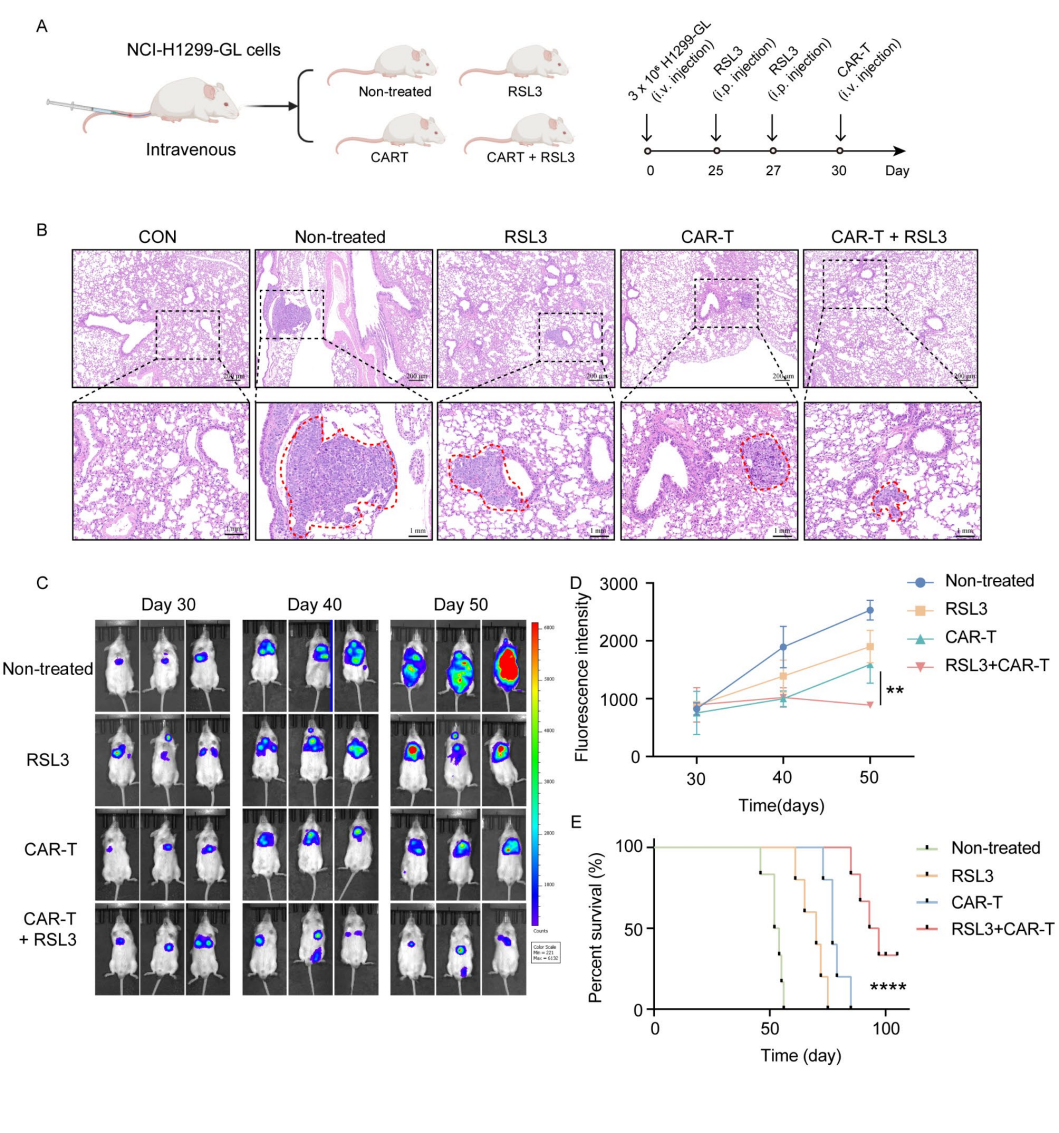
ROR1 CAR-T cells inhibit tumor growth in a metastatic mouse model of human NSCLC. (**A**) Schematic of the combination treatment in a human NSCLC metastatic mouse model, established by injecting NCI-H1299-GL cells into NCG mice. (**B**) Hematoxylin and eosin staining of the NSCLC metastatic model, ×5 (top), ×20 (bottom). (**C**–**D**) Time-lapse luciferase imaging of ROR1 CAR-T cells after RSL3 or vehicle treatment (*n* = 6). (**E**) NSCLC model mice survival curve following RSL3 and CAR-T treatment (*n* = 6). Statistical significance: n.s. (*p* > 0.05); *p < 0.05; **p < 0.01; ****p < 0.0001

To evaluate the therapeutic potential of ROR1 CAR-T cells combined with RSL3, we developed a human metastatic NSCLC mouse model (Fig. 5A). Tumor progression was tracked using a luciferase-based imaging system. Hematoxylin and eosin staining confirmed the colonization of tumor cells in the lungs, and combined therapy demonstrated noticeable effects by day 50 (Fig. 5B). The combination of ROR1 CAR-T cells and RSL3 exhibited significantly greater therapeutic efficacy in attenuating metastatic progression compared with the other three treatment groups (Fig. 5C and D). Additionally, this combined approach significantly extended the survival of treated mice compared with those receiving either RSL3 or ROR1 CAR-T treatment alone (Fig. 5E). Taken together, the combination therapy effectively targets metastatic sites, such as the lungs, and significantly improves survival outcomes in this NSCLC metastasis model.

## Discussion

ROR1 is a member of the receptor tyrosine kinase-like orphan receptor family. We confirmed membrane localization of ROR1 in patients with recurrent and treatment-refractory disease, which is the primary target population for CAR-T therapy. ROR1 has emerged as a promising target in cancer immunotherapy because of its restricted expression in normal adult tissues and its overexpression in various malignancies. Over the past decade, the role of ROR1 in cancers has been investigated, particularly in chronic lymphocytic leukemia, breast cancer, ovarian cancer, and NSCLC [23, 35, 36]. Notably, targeting ROR1 can effectively reduce tumor growth, making it an attractive candidate for CAR-T cell therapy. While the clinical translation of ROR1-targeted therapies for solid tumors remains in early development, its success in hematological malignancies, such as leukemia [37], has provided critical information for its potential application for more challenging solid tumor types. These advances highlight a promising future for ROR1-targeted CAR-T cell therapies, especially in patients with recurrent, treatment-refractory disease.

Combinatorial strategies are showing promise for enhancing CAR-T cell therapy for solid tumors, where immune evasion, limited T cell trafficking, and immunosuppressive microenvironments hinder efficacy [38, 39]. A key challenge is the treatment of “cold tumors,” which have low immune infiltration and activation. Combinatorial approaches aim to reprogram the tumor microenvironment, converting cold tumors into “hot” ones by targeting multiple pathways involved in relapse and tumor evasion [15]. Shi et al. found that combining lenalidomide with CD19 CAR-T cells increased IL-2 and IFN-γ production and improved overall response rates in multiple myeloma patients [40]. Therefore, we aimed to screen for drugs that synergize with CAR-T therapy to enhance the killing of lung cancer cells.

We screened numerous ferroptosis inducers to identify drugs that can overcome defective tumor cell death. RSL3 and ML210 were identified as agents that can sensitize tumor cells to undergo ferroptosis and lead to a more robust anti-tumor response. Recently, pharmaceutical companies, including Kojin Therapeutics, have investigated the potential therapeutic effects of compounds such as RSL3 and ML210 on tumors. These companies are conducting pertinent clinical studies with the goal of translating research findings into practical medical applications. However, it remains unclear whether activating endogenous ferroptosis mechanisms can restrict tumor progression and modulate the sensitivity of CAR-T cell therapy. We demonstrated ACSL4 to be the critical downstream mediator of IFN-γ, via its activation of endogenous ferroptotic mechanisms through metabolic lipid reprogramming. ACSL4 preferentially uses polyunsaturated fatty acids (PUFAs), including arachidonic acid, as substrates [41]. It activates arachidonic acid by catalyzing its thioesterification with coenzyme A (CoA) to produce arachidonoyl-CoA, the first step in arachidonic acid metabolism [42].

IFN-γ from activated CAR-T cells, in conjunction with PC-PUFA2, directly induces ferroptosis in tumor cells, serving as a key mechanism for cytotoxic T-lymphocyte-mediated tumor cell killing. Our findings confirm that PC-PUFA2 is a pro-ferroptosis lipid marker that plays a crucial role in this process.

Oxidized phosphatidylethanolamines with arachidonoyl and adrenoyl tails are a unique feature of ferroptotic cells [43, 44], while PC-PUFA2 is a proximal lipid effector that drives ferroptosis. As a potent downstream effector of dietary PUFAs, PC-PUFA2 modulates oxidative stress by disrupting mitochondrial ROS homeostasis [29, 45]. Consequently, PC-PUFA2 represents a critical driver of ferroptosis, which offers possibilities for the detection and regulation of disease states.

Despite these promising findings, several considerations should be taken into account. While this study primarily focused on ROR1-expressing NSCLC, the therapeutic efficacy of this combination approach in other ROR1-positive malignancies, such as ovarian or breast cancer, remains to be explored. Our results indicate that ROR1 is highly expressed in ICB-resistant NSCLC tumors, although its exact role in the resistance mechanism requires further clarification. Additionally, although combination therapy leads to increased ROS levels and ferroptosis in tumor cells, the impact of this approach on other immune cells in the immune microenvironment (such as macrophages or regulatory T cells) has not been investigated, which could potentially influence the overall therapeutic efficacy. Future research should aim to optimize the therapeutic regimen for ROR1-targeted therapy, such as determining the most effective dosing schedules and delivery methods. Additionally, leveraging advanced technologies, such as single-cell sequencing, may provide deeper insights into the tumor microenvironment’s response to treatment.

## Conclusions

Combining ROR1 CAR-T cells with ferroptosis inducers enhances anti-tumor efficacy in NSCLC by promoting ferroptosis through increased lipid peroxidation. Importantly, overcoming resistance to IFN-γ signaling is key to improving the efficacy of CAR-T cell therapy targeting solid tumors. Our study highlights the transformative potential of ROR1-targeted combination therapies in overcoming ICB resistance in NSCLC. Our findings also provide a promising new avenue for more potent and effective cancer treatments that can potentially be extended to other solid tumor malignancies, either as a stand-alone treatment or in combination with other therapeutic modalities.

## Electronic supplementary material

Below is the link to the electronic supplementary material.

Supplementary Material 1

Supplementary Material 2 The link below is not available to download.

## Data Availability

No datasets were generated or analysed during the current study.

## Abbreviations

CAR-T cells: Chimeric Antigen Receptor T cells
PC-PUFA2: Phosphatidylcholine with Diacyl-Polyunsaturated Fatty Acid Tails
NSCLC: Non-Small Cell Lung Cancer
ROR1: Receptor Tyrosine Kinase-Like Orphan Receptor 1
ROS: Reactive Oxygen Species
IFN-γ: Interferon Gamma
ACSL4: Acyl-CoA Synthetase Long Chain Family Member 4
ICB: Immune Checkpoint Blockade
TCGA: The Cancer Genome Atlas
RSL3: (1S,3R)-RSL3
Fer-1: Ferrostatin-1
TNF-α: Tumor Necrosis Factor Alpha
TfR1: Transferrin Receptor 1 (TfR1)
NCG: NOD/ShiLtJGpt-*Prkdc*^em26Cd52^*Il2rg*^em26Cd22^/Gpt
SEM: Standard Error of Mean
scFv: Single-Chain Fragment Variable
GFP: Green Fluorescent Protein
GPX4: Glutathione Peroxidase 4
MFI: Relative mean fluorescence intensity
