# Supplementary material for: ROR1 CAR-T cells and ferroptosis inducers orchestrate tumor ferroptosis via PC-PUFA2
Source: Biomark Res. 2025 Jan 23;13:17. doi: 10.1186/s40364-025-00730-0 (PMC11756136; doi:10.1186/s40364-025-00730-0)
Supplement: Supplementary file 1 — Supplementary Material 1 [file 40364_2025_730_MOESM1_ESM.docx]

**Supplementary Tables**

**Table S1** sgRNA sequence

| **Name** | **sgRNA sequence (5'to3')** |
| --- | --- |
| ACSL4-sgRNA-1 | ACCACTAGTATACATAACAA |
| ACSL4-sgRNA-2 | GAATTATCTTACCCCAGTCC |
| IFNGR1-sgRNA-1 | GGTACTCCCAATATACGATA |
| IFNGR1-sgRNA-2 | AGGATACTGGAATCGCTAAC |

**Table S2** Clinical information of patients with NSCLC

| **Characteristics** | **Patients (n=10)** |
| --- | --- |
| Age [year (range)] | 61.3 (36-75) |
| Gender (%) |  |
| Male | 60 |
| Female | 40 |
| Patient ethnicity (%) |  |
| Asian | 100 |
| Other | 0 |
| Tumor size (cm) |  |
| Mean ± standard | 3.19 ± 0.54 |
| Range | 2.7- 4.5 |
| Histology (%) |  |
| NSCLC | 100 |
| Other | 0 |
| Immunotherapy (%) |  |
| PD-1 | 80 |
| PD-L1 | 20 |

**Table S3** Relative quantification of PC (22:6_22:6)-CH3

|  | Non-treated  (n=5) | RSL3-treated  (n=5) | CAR T-treated  (n=5) | CAR T+RSL3  (n=5) |
| --- | --- | --- | --- | --- |
| PC (22:6_22:6)-CH3 | 8796590.60675 | 13147255.14125 | 8598837.71325 | 16808560.663 |

PC: Phosphatidylcholine
