# Supplementary material for: ROR1 CAR-T cells and ferroptosis inducers orchestrate tumor ferroptosis via PC-PUFA2
Source: Biomark Res. 2025 Jan 23;13:17. doi: 10.1186/s40364-025-00730-0 (PMC11756136; doi:10.1186/s40364-025-00730-0)
Supplement: Supplementary file 2 — Supplementary Material 2 The link below is not available to download. [file 40364_2025_730_MOESM2_ESM.docx]

**Supplementary Figures**


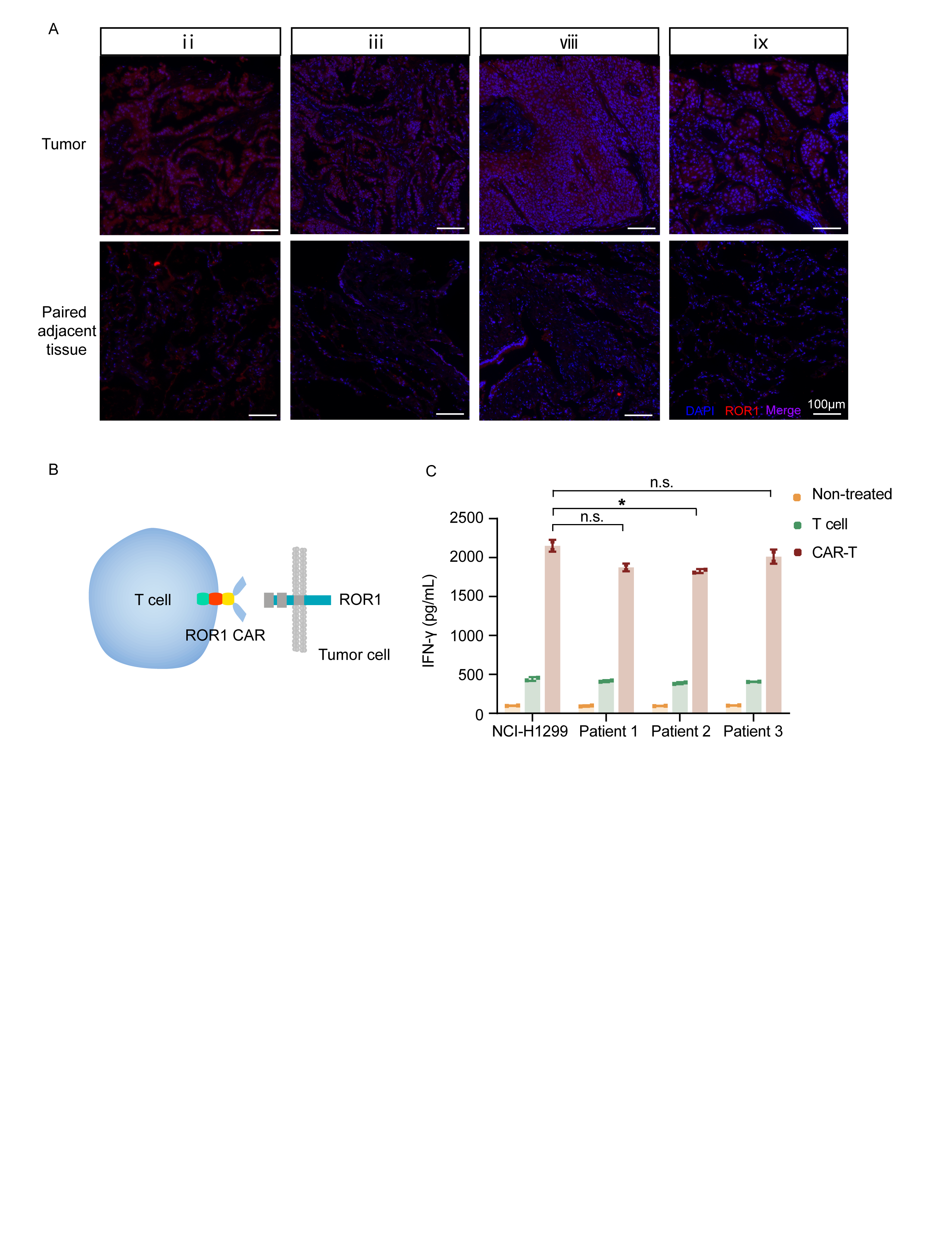


Figure S1. Evaluation of ROR1 as a Therapeutic Target in Recurrent Lung Cancer Patients.

(A) ROR1 expression in tumor tissues from recurrent NSCLC patients. (B) Schematic representation of the ROR1 CAR-T against tumor cells. (C) Levels of IFN-γ in the supernatants of co-cultured ROR1 CAR-T and H1299 cells, measured by ELISA. Statistical significance: n.s. (*p* > 0.05); **p* < 0.05; ***p* < 0.01; *****p* < 0.0001.


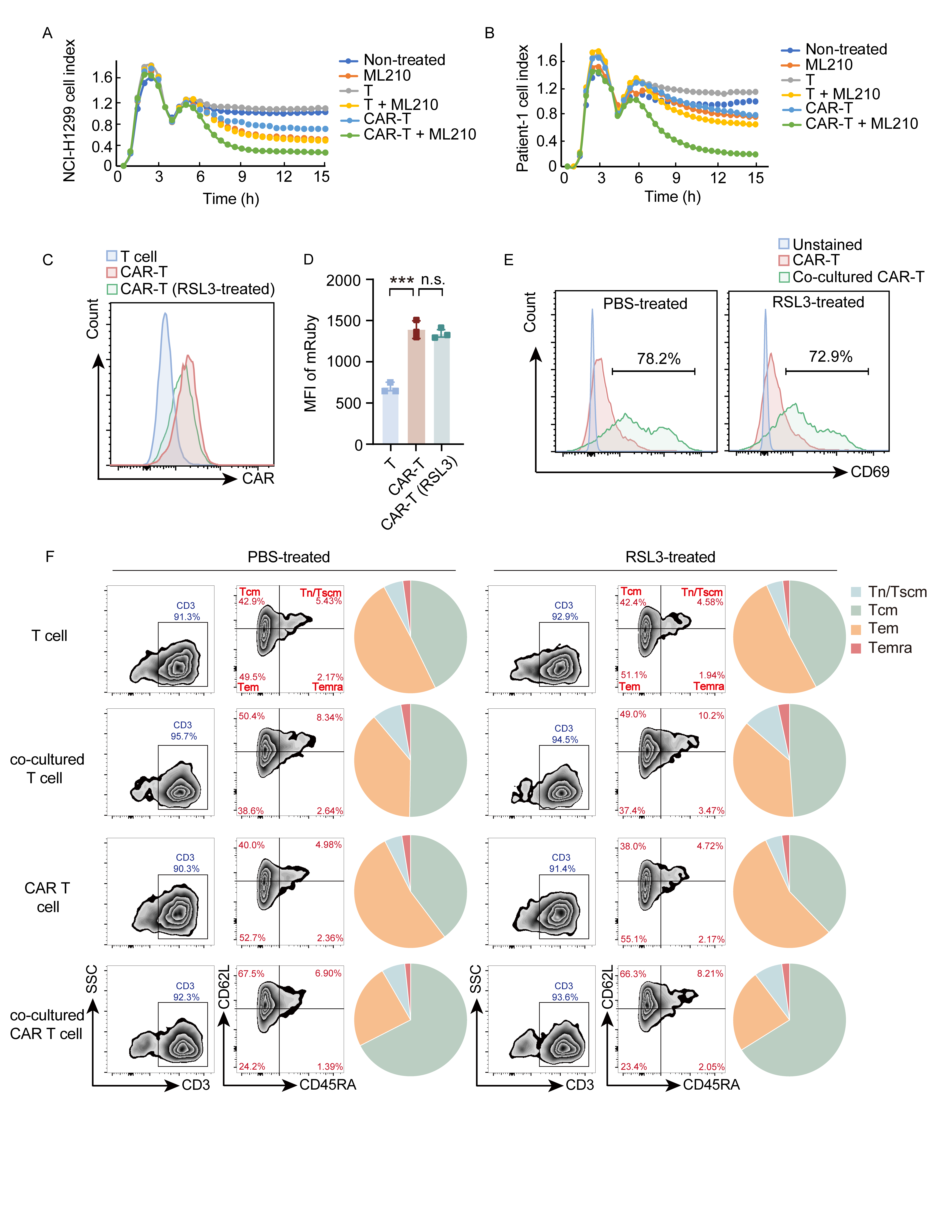


Figure S2. Validation of Key Drugs Impacting CAR-T Cell Cytotoxicity in NSCLC.

(A-B) Combination treatment with 2μM ML210 enhances the cytotoxicity of ROR1 CAR-T cells against NCI-H1299 and primary cells. (C-D) The mRuby fluorescence signal was detected by flow cytometry for determination of CAR-T cell positivity. (E) CD69, a marker for the activation of CAR T cells, was detected by flow cytometry after coculture with tumor cells, with or without RSL3 treatment. (F) CD45 and CD62 cells were analyzed from CD3 positive cell populations cocultured with tumor cells, with or without RSL3 treatment. Statistical significance: n.s. (*p* > 0.05); **p* < 0.05; ***p* < 0.01; *****p* < 0.0001.


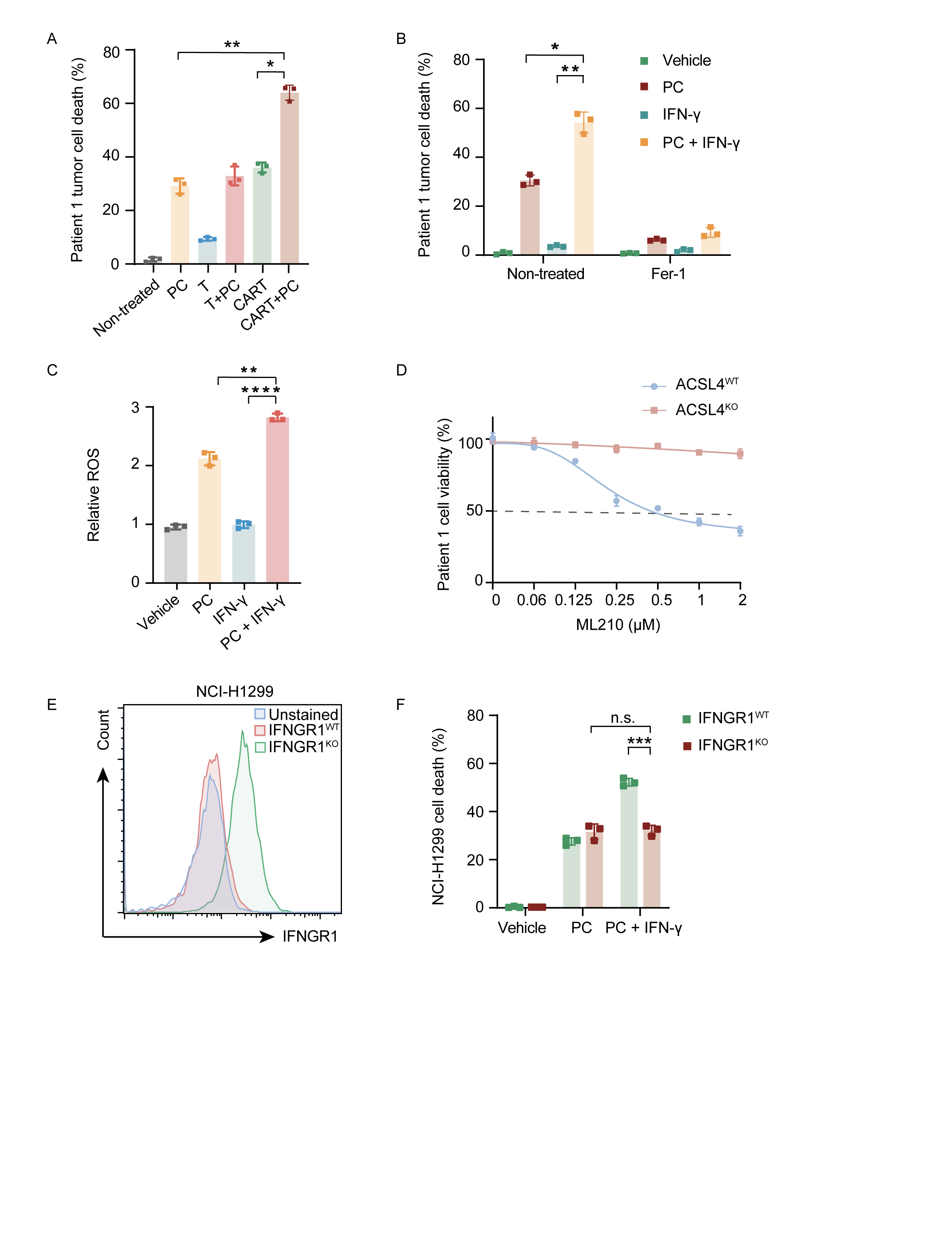


Figure S3. ROR1 CAR-T and RSL3 cooperatively induce tumor cell ferroptosis via PC-PUFA2.

(A) Percentage of patient-1 death after 24 h co-culture with ROR1 CAR-T cells or untransduced T cells with or without 50 μM PC-PUFA2. (B) Patient-1 treated with vehicle, 10 ng/ml IFN-γ, or 50 μM PC-PUFA2 with or without 10 μM Fer-1. Cell death was measured after 24 h. (C) MFI of oxidized C11-BODIPY 581/591 compared with vehicle, presented as mean ± SEM from three biological replicates. (D) Cell viability of NCI-H1299 ACSL4^WT^ or ACSL4^KO^ cells treated with 2μM ML210 at the indicated concentrations after 10 h. (E) IFNGR1 knockout efficiency was detected by flow cytometry. (F) Cell death of NCI-H1299 IFNGR1^wt^ and NCI-H1299 IFNGR1^KO^ cells treated with 50 μM PC-PUFA2 with or without 10 ng/ml IFN-γ for 24 h (n = 3). Statistical significance: n.s. (*p* > 0.05); **p* < 0.05; ***p* < 0.01; *****p* < 0.0001.
